# Enhanced Solar Light Photocatalytic Activity of Ag Doped TiO_2_–Ag_3_PO_4_ Composites

**DOI:** 10.3390/nano10040795

**Published:** 2020-04-21

**Authors:** Abdessalem Hamrouni, Hanen Azzouzi, Ali Rayes, Leonardo Palmisano, Riccardo Ceccato, Francesco Parrino

**Affiliations:** 1 Laboratoire de Recherche Catalyse et Matériaux pour l’Environnement et les Procédés URCMEP (UR11ES85), Faculté des Sciences de Gabès, Université de Gabès, Campus Universitaire Cité Erriadh, Gabès 6072, Tunisia; hamrouni-28@hotmail.fr (A.H.); hanenazzouzi408@gmail.com (H.A.); ali.rayes@fsb.rnu.tn (A.R.); 2 Department of Engineering, University of Palermo, Viale delle Scienze, Ed. 6, 90128 Palermo, Italy; leonardo.palmisano@unipa.it; 3 Department of Industrial Engineering, University of Trento, Via Sommarive 9, 38123 Trento, Italy; riccardo.ceccato@unitn.it

**Keywords:** Ag@TiO_2_–Ag_3_PO_4_ heterojunction, solar photocatalysis, 4–nitrophenol degradation, sol–gel synthesis

## Abstract

Composites comprised of Ag_3_PO_4_ and bare TiO_2_ (TiO_2_@Ag_3_PO_4_) or silver doped TiO_2_ (Ag@TiO_2_–Ag_3_PO_4_) have been synthesized by coupling sol–gel and precipitation methods. For the sake of comparison, also the bare components have been similarly prepared. All the samples have been characterized by X-ray diffraction (XRD), UV-vis diffuse reflectance spectroscopy (DRS), scanning electron microscopy (SEM), Fourier transformed infrared spectroscopy (FTIR), photoelectrochemical measurements, and specific surface area (SSA) analysis. The optoelectronic and structural features of the samples have been related to their photocatalytic activity for the degradation of 4–nitrophenol under solar and UV light irradiation. Coupling Ag_3_PO_4_ with silver doped TiO_2_ mitigates photocorrosion of the Ag_3_PO_4_ counterpart, and remarkably improves the photocatalytic activity under solar light irradiation with respect to the components, to the TiO_2_–Ag_3_PO_4_ sample, and to the benchmark TiO_2_ Evonik P25. These features open the route to future applications of this material in the field of environmental remediation.

## 1. Introduction

Each year, the surface of Earth receives 3850000 EJ of solar energy. However, only 0.014% of it is currently exploited to cover the energy demand, and to face environmental issues [1]. In fact, efficient solar light conversion is one of the hot topics of our society, which is finally aware of the need to develop and actuate sustainable processes to mitigate the dramatic climate changes and environmental disaster we are the witness of in the last years. The unique properties of visible light active semiconductors allow one to use them to convert solar energy into photogenerated charges, which, in turn, enable electricity generation or trigger useful chemical reactions. In this way, it is possible to mimic nature by storing solar energy in chemical bonds, by producing high value-added chemical compounds in a “green” way, or by removing hazardous compounds through their photoinduced mineralization. Titanium dioxide (TiO_2_) has been widely investigated and used to this aim, mainly due to its remarkable photoactivity, low cost, and robustness. However, TiO_2_ can be only activated upon UV light irradiation, which represents only ca. 5% of the solar light spectral emission. In order to overcome this limitation, TiO_2_ has been variously modified to extend its light absorption range toward the visible light region [2]. In many cases sensitization of TiO_2_ under visible light is achieved by modifying its surface with dyes [3], chromogenic species [4], or metal nanoparticles [5]. However, the resulting materials are often poorly stable, with few exceptions [6], and mainly useful for niche applications rather than for environmental remediation, where large effluent volumes need to be treated. Bulk modification, instead, provides more stable materials [7]. However, the influence of bulk defectivity on the recombination rate of the photogenerated charges, and in turn on the photocatalytic activity, is still object of scientific debate [8] due to the highly specific nature of the photoactive materials and of their interaction with light [9]. Alternatively, intrinsically visible light active semiconductors such as oxides [10], chalcogenides [11], and mixed semiconductors [12,13,14,15,16] have been recently investigated to trigger photocatalytic reactions under visible light irradiation [17]. Among them, the p-type semiconductor silver orthophosphate (Ag_3_PO_4_) has been often reported as a promising alternative to visible light active TiO_2_-based materials in the field of solar photocatalysis. The narrow band gap energy enables its visible light activation, and the strong oxidizing power [18,19,20] justifies its extensive investigation in scientific reports dealing with organic dye degradation, bacterial disinfection, and water splitting reaction [18,19,21]. In particular, quantum efficiency up to 90% can be achieved for O_2_ evolution under visible light irradiation in the presence of Ag_3_PO_4_. Worth of mention is also the great versatility of Ag_3_PO_4_, whose morphology can be easily tuned by opportunely adjusting the synthesis procedure. For instance, Zwara et al. [22] recently reported the synthesis of variously shaped Ag_3_PO_4_ samples with high photoactivity for the degradation of phenol under visible light. However, Ag_3_PO_4_ is prone to photocorrosion because photogenerated electrons easily reduce lattice silver ions to metallic silver eventually destroying the photocatalyst after few working cycles [23]. The photostability of Ag_3_PO_4_ can be enhanced in various ways. Some authors proposed the use of sacrificial reducing agents to hinder photocorrosion. However, this reduces the energy stored in the bonds of the products, which is particularly detrimental for water splitting [24] or for synthetic applications [25]. A much suitable approach consists in coupling different semiconductors [12,13,14,15,16]. To this aim, p–n heterostructures of Ag_3_PO_4_ and TiO_2_ are particularly promising. Yao et al. [26] reported the enhanced visible light photocatalytic activity of Ag_3_PO_4_ nanoparticles deposited onto commercial TiO_2_ (Evonik P25). In this case, degradation of dyes such as methylene blue and rhodamine B have been used as model visible light reactions, so that it is difficult to assess if a direct or indirect photocatalytic process is taking place [27]. However, results were confirmed by Rawal et al. [28], which used a homemade polycrystalline TiO_2_ coupled with Ag_3_PO_4_ for the visible light degradation of 2–propanol in gas phase. Teng et al. [29] reported the synthesis of ordered TiO_2_ nanotubes decorated with Ag_3_PO_4_ and Ag nanoparticles, and tested the composites for the removal of 2–chlorophenol in aqueous solution. Silver nanoparticles, deposited on the surface of Ag_3_PO_4_, acted as electron sinks thus preventing photocorrosion [30,31]. However, in the latter case, the photocatalyst is a ternary composite (Ag–Ag_3_PO_4_–TiO_2_) presenting exposed silver nanoparticles, which can aggregate and be easily removed during real waste water treatments. With the aim of simplifying the system for environmental applications, by providing higher robustness and simultaneously maintaining visible light absorption and high photoactivity, in the present investigation we introduced silver into the lattice of TiO_2_ and designed a binary heterojunction between Ag_3_PO_4_ and nanostructured Ag@TiO_2_. This composite provides efficient charge separation due to the unique optoelectronic features of the Ag@TiO_2_ counterpart, and high photocatalytic activity under solar light irradiation. In particular, the photocatalytic degradation of 4–nitrophenol (4–NP) under UV and natural solar light irradiation have been investigated, and the activity results have been related to the physico-chemical features of the samples. 

## 2. Materials and Methods 

### 2.1. Synthesis of the Samples

Ag_3_PO_4_ was synthesized according to a simple precipitation protocol. Silver nitrate (AgNO_3_, Park scientific, Northampton, UK 99.8%) and monosodium phosphate (NaH_2_PO_4_∙2H_2_O, Merck, Darmstadt, Germany, extra pure) salts were employed as sources of Ag^+^ and PO_4_^3−^ ions, respectively. Two aqueous solutions of Ag^+^ (0.1 M) and PO_4_^3−^ (0.3 M) were prepared separately, and 246 mL of the silver solution were added dropwise to 330 mL of the orthophosphate solution in order to provide large excess of the PO_4_^3−^ anions. The obtained yellow precipitate was separated by centrifuge, washed several times with distilled water, and finally dried at 80 °C overnight.

TiO_2_ was synthesized by dissolving 11.3 mL of titanium isopropoxide (Ti[OCH(CH_3_)_2_]_4_, Sigma Aldrich, St. Louis, MO, USA, 97%) in 11.4 mL isopropanol (C_3_H_7_OH, 99.8%, Sigma Aldrich, St. Louis, MO, USA). Hydrolysis started by adding 2.15 mL of acetic acid (CH_3_COOH, Sigma Aldrich, St. Louis, MO, USA, glacial 99.5%) and 1.35 mL distilled water, corresponding to molar ratio n_H_2___O_/n_Ti_ equal to 2. The resulting solution was stirred at 70 °C for about 20 min, until a white gel was obtained. The gel was then dried at 100 °C overnight and the resulting powder was calcined at 400 °C for 3 h in static air.

Silver doped TiO_2_ (Ag@TiO_2_) was synthesized following the same procedure used for bare TiO_2_, but dissolving in the starting isopropanol solution an opportune amount of silver nitrate to reach an Ag/Ti molar ratio equal to 0.01. Notably, the percentage of silver used in this work is the optimum one in terms of photocatalytic activity and stability, as demonstrated in previous reports [32,33,34,35,36,37].

The composites TiO_2_–Ag_3_PO_4_ and Ag@TiO_2_–Ag_3_PO_4_ were synthesized by dispersing the as prepared TiO_2_ and Ag@TiO_2_, respectively, in the 0.1 M monosodium phosphate aqueous solution just before the addition of the 0.1 M AgNO_3_ solution, and by following the same procedure described for the preparation of the Ag_3_PO_4_ sample. The molar percentage of TiO_2_ (bare and silver doped) and Ag_3_PO_4_ was 25% and 75%, respectively, in both the composites. All the above mentioned chemicals were used as received without further purification.

### 2.2. Characterization 

X-ray diffraction (XRD) analysis of the samples has been performed by using a Rigaku D-Max III powder diffractometer, Tokyo, Japan, in Bragg–Brentano geometry, working between 10 and 70° (2θ range) with sampling step of 0.05° and a counting time of 2 s. Monochromatic Cu Kα radiation was used, operating at a voltage of 40 kV and a current of 30 mA; a curved graphite monochromator was inserted in the diffracted beam. Both quantitative analysis and crystallite size determination were performed by means MAUD software^®^ (Version 2.26, by Luca Lutterotti, University of Trento, 2010) [38,39], by combining the Rietveld-based method for the determination of weight percentages of phases, and line profile analysis based on the Warren–Averbach theory for the evaluation of mean crystallite sizes (D) and microstrains (ε).

UV-visible diffuse reflectance spectra were recorded by a Perkin Elmer Lambda 950 UV-vis spectrophotometer, Waltham, MS, USA, equipped with an integrating sphere, in the range of 190–800 nm and by using barium sulfate (BaSO_4_, Sigma Aldrich, St. Louis, MO, USA, p.a.) as the reference. Reflectance (R_∞_) was converted by the instrument software to F(R_∞_) values according to the Kubelka–Munk theory. The bandgap was obtained from a plot of [F(R_∞_)hν]^1/2^ vs. the energy of the exciting light, by assuming that all of the photocatalysts are indirect semiconductors. The band gap energy (E_g_) values were determined by extrapolating the linear part of the plot to the x axis.

Scanning electron microscopy (SEM) and energy dispersive X-Ray spectroscopy (EDS) analyses were carried out using a field emission-scanning electron microscope (FE-SEM, Supra 40/40VP, Zeiss, Oberkochen, Germany), operating at a voltage of 20 kV on specimens upon which a thin layer of Pt/Pd had been deposited under Ar atmosphere. Fourier transformed infrared spectroscopy (FTIR) was carried out on a Thermo Nicolet Avatar 330 FTIR, Thermo Fisher, Waltham, MS, USA, spectrophotometer.

To determine the specific surface area (SSA) and the pore size distribution (PSD) of the photocatalysts, nitrogen adsorption measurements were performed at the liquid nitrogen temperature by using a Micromeritics, Norcross, GA, USA, ASAP 2010 porosimeter. All the samples were degassed below 1.3 Pa at 200 °C prior to the measurement. SSA’s were calculated by the Brunauer−Emmet−Teller (BET) equation in the P/P^0^ interval 0.05–0.33. PSD’s were calculated by using the Barrett Joyner Halenda (BJH) method applied both on the adsorption and desorption branches of the isotherms.

Determination of the flat band potential of selected samples was performed in a Pyrex round bottom flask (V = 150 mL) irradiated with a 500 W medium pressure mercury lamp. 100 mg of the photocatalyst were dispersed into 100 mL of a 0.1 M KNO_3_ solution. The working electrode was a platinum foil and an Ag/AgCl electrode was used as the reference. The suspension was irradiated under nitrogen bubbling for 30 min and then 20 mg of MV^2+^ (1,1′–dimethyl–4,4′–bipyridinium dichloride) or DP^2+^ (4,5–dihydro–3a,5a–diaza–pyrene dibromide) were added as the electron scavengers. A pH meter Thermo Orion (Thermo Scientific, Waltham, MS, USA 720A and a multimeter Metex 3800 (Metex Corp., Seoul, South Korea) were used to measure the values of pH and the potential (V), respectively. Potentiometric titration was carried out by adjusting the pH with HNO_3_ and NaOH solutions. 

### 2.3. Photocatalytic Experiments

A Pyrex tubular photoreactor (V = 100 mL) was used for the photodegradation tests of 4–nitrophenol (4–NP, Sigma Aldrich, St. Louis, MO, USA, 98%). In a representative run the reactor contained 80 mL of an aqueous solution of 4–NP at a concentration of 10 mg∙L^−1^ in which 80 mg of the photocatalyst were dispersed. The catalyst amount of 1 g∙L^−1^ enabled the safe comparison of the results obtained in the presence of different samples. A magnetic stirrer ensured the homogeneity of the reaction mixture. The suspension was kept in the dark under agitation during 60 min prior to irradiation, in order to reach the adsorption–desorption equilibrium. Samples were taken at different time intervals and filtered using a 0.2 μm Millipore filter to separate the photocatalyst. The concentration of 4–NP during irradiation time was measured by using a Perkin Elmer 950 UV-vis spectrophotometer, Waltham, MS, USA. Tests under UV light irradiation were performed by using six Philips (Amsterdam, The Netherlands) FSL BL T8 lamps (10 W each) axially positioned around the photoreactor, with the main emission peak at 365 nm. The radiation power absorbed per unit volume of the suspension was about 0.76 mW∙mL^−1^ measured by a Delta Ohm photo quantum meter (model HD9021) (Selvazzano Dentro, Italy) with a LP 9021 UVA sensor probe. 4–NP photodegradation tests under solar light were carried out by exposing to natural sun light at the same time as many different reactors in the presence of the investigated samples, during a sunny day in Gabes (Tunisia, 33°53′24″N 10°06′36″E) between 11 am and 4 pm in June. This approach allows one to safely compare the photocatalytic activity of the samples under natural solar light, as all of the photoreactors worked under the same irradiation conditions. The photocatalytic results have been compared with those obtained in the presence of commercial TiO_2_ P25 (Evonik). The mean sun light intensity during the solar photocatalytic runs was 8.2 W∙m^−2^ in the range 315–400 nm, and 2800 μE∙m^−2^∙s^−1^ in the range 400–700 nm.

## 3. Results

### 3.1. Structure and Morphology of the Samples

**Figure 1:**
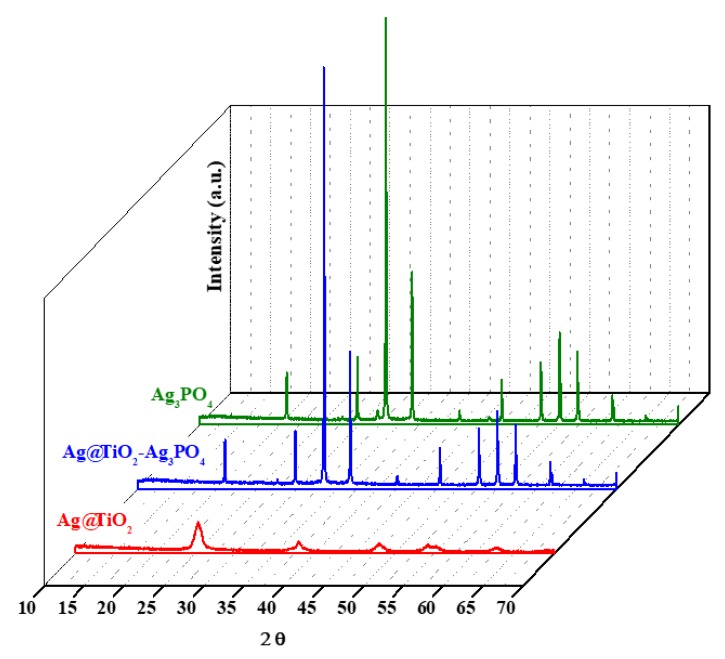
XRD patterns of Ag_3_PO_4_, Ag@TiO_2_, and Ag@TiO_2_–Ag_3_PO_4_ composites.

Figure 1 reports the XRD patterns of Ag_3_PO_4_, Ag@TiO_2_, and Ag@TiO_2_–Ag_3_PO_4_ samples. The XRD patterns of TiO_2_ and of TiO_2_–Ag_3_PO_4_ are reported in the Supporting Information (Figures S1–S4), being similar to those of Ag@TiO_2_ and Ag@TiO_2_–Ag_3_PO_4_, respectively.

Ag_3_PO_4_ presents body centered cubic structure (JCPDS N°06-0505) while bare TiO_2_ shows the typical patterns of the anatase structure (JCPDS N°21-1272). Ag_3_PO_4_ signals were sharper with respect to those of TiO_2_ indicating the lower crystallinity of the latter, typical of powders obtained from the sol–gel method. The Ag@TiO_2_ sample presents qualitatively the same patterns of the TiO_2_ sample indicating that the anatase structure is not affected upon silver doping (Ag/Ti molar ratio = 0.01). Moreover, no traces of impurities ascribable to silver containing compounds such as oxides can be observed, suggesting the successful incorporation of silver into the TiO_2_ lattice. On the basis of XRD data, the lattice parameters (a, b, and c) and the mean crystallites dimension (D) were calculated for the pure components as summarized in Table 1, which also reports the microstrain values (ε), the specific surface area (SSA), and the pores volume (V_p_).

By comparing the lattice parameters and the crystallite size of TiO_2_ and Ag@TiO_2_ in Table 1, it is evident that in the presence of silver the unit cell shrinked along c and became larger along a and b directions. These changes, due to an isotropic size dependent effect [40], can be attributed to the insertion of substitutional silver ions into the TiO_2_ lattice. In fact, the bigger size of Ag^+^ (1.26 Å) with respect to that of Ti^4+^ ions (0.60 Å) modifies the lattice parameter and influences the crystal growth and the particle size. Literature reports suggest that silver doping generally hinders the growth of crystallites [41] due to grain boundary restraining caused by symmetry-breaking effects of the dopant [36]. At the same time, a decrease in crystallite dimensions is associated to an increase of microstrain values, reported in Table 1, due to the presence of surface tensions that provoke an increase of stresses [42]. This is confirmed also in this work, being the size of particles slightly smaller in the silver doped TiO_2_ with respect to the bare sample. Similar effects have been observed in literature when doping metal oxides with other metal ions [43].

The specific surface area of Ag_3_PO_4_ sample is much lower than that of the TiO_2_-based ones, in agreement with its higher crystallinity observed by XRD analysis. The mixed samples present intermediate specific surface area, closer to that of Ag_3_PO_4_, which is present in a higher amount with respect to TiO_2_. 

**Figure 2:**
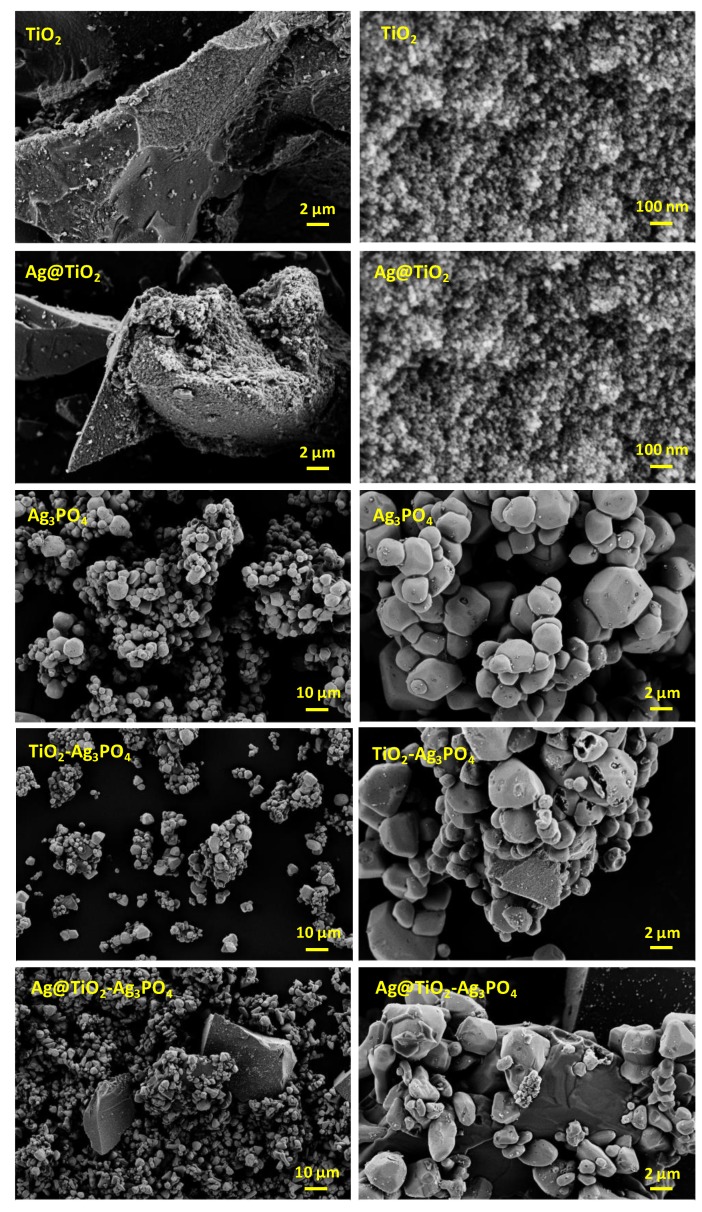
SEM images of TiO_2_, Ag@TiO_2_, Ag_3_PO_4_, TiO_2_–Ag_3_PO_4_, and Ag@TiO_2_–Ag_3_PO_4_ samples.

The morphology and the composition of the samples were investigated by using scanning electron microscopy (SEM). Figure 2 shows the SEM images of all the samples. EDS analyses, reported in the Supporting Information (Figure S5a–c), confirm the presence of the expected atoms in percentage relatively close to the estimated one. No impurities containing foreign atoms could be detected. Images of TiO_2_ and Ag@TiO_2_ samples do not present significant differences, and are composed of micrometric agglomerates comprised of small nanoparticles of few tens of nanometers. This is in agreement with the crystallites dimension retrieved by XRD analysis. On the other hand, the Ag_3_PO_4_ particles are in the range of 1 to 5 micrometers and appear quite homogeneous in morphology and size. The composites TiO_2_–Ag_3_PO_4_ and Ag@TiO_2_–Ag_3_PO_4_ are comprised, respectively, of large TiO_2_ or Ag@TiO_2_ agglomerates upon which Ag_3_PO_4_ particles can be observed.

**Figure 3:**
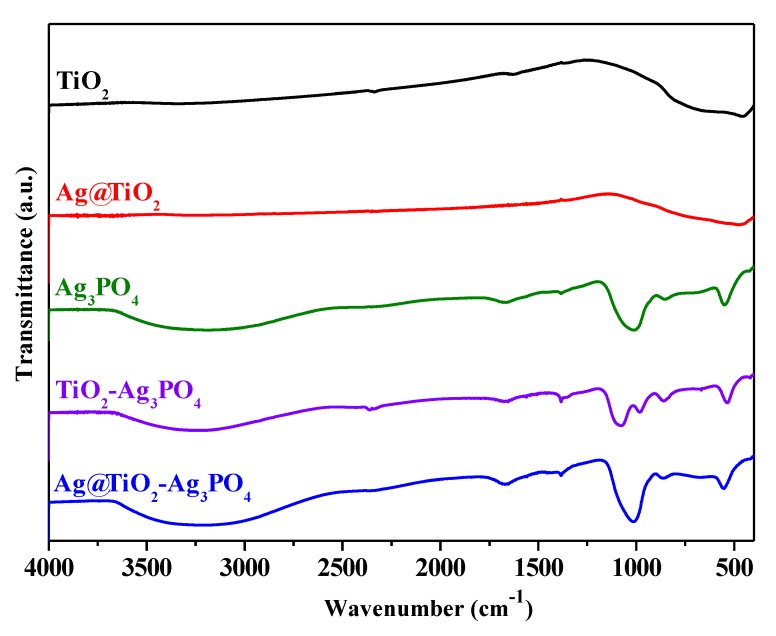
FTIR spectra of TiO_2_ (black spectrum), Ag@TiO_2_ (red spectrum), Ag_3_PO_4_ (green spectrum), TiO_2_–Ag_3_PO_4_ (purple spectrum), and Ag@TiO_2_–Ag_3_PO_4_ (blue spectrum) samples.

Infrared spectra of the samples are shown in Figure 3. 

The spectra of the Ag_3_PO_4_ containing samples present a signal at 1649 cm^−1^ and a broad band between 3000 and 3600 cm^−1^ that can be attributed to the bending and to the stretching of O–H groups of adsorbed water, respectively [44,45]. The characteristic peaks of the PO_4_^3−^ groups are present at 1010 cm^−1^ and 558 cm^−1^ corresponding to P–O stretching vibrations [44,45]. The IR spectra of TiO_2_ and Ag@TiO_2_ present a broad band between 500 and 900 cm^−1^, ascribable to Ti–O vibrations [46].

### 3.2. Optical Properties

**Figure 4:**
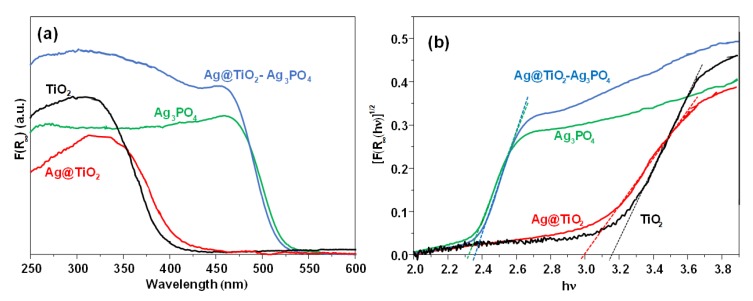
(**a**) UV-vis absorption spectra and (**b**) Tauc plot of the TiO_2_ (black line), Ag@TiO_2_ (red line), Ag_3_PO_4_ (green line), and Ag@TiO_2_–Ag_3_PO_4_ (blue spectrum) samples.

The absorption spectra of the samples are presented in Figure 4a. The values of F(R_∞_), calculated according to the Kubelka–Munk theory from the values of diffuse reflectance, were proportional to the absorbance. By considering that all of the samples are indirect crystalline semiconductors [47], the band gap energy can be obtained by extrapolating the linear part of a plot of [F(R_∞_)hν]^1/2^ vs. the energy of the exciting light (Figure 4b). 

The adsorption edge of the Ag_3_PO_4_ containing samples is at about 540 nm due to the narrow band gap of this semiconductor. The conduction band of Ag_3_PO_4_ presents Ag 5s and 5p character, while the valence band is composed of Ag 4d and O 2p orbitals [48,49]. As expected, Ag@TiO_2_–Ag_3_PO_4_ sample presented lower absorption in the visible region with respect to bare Ag_3_PO_4_, but higher absorption in the UV region due to the typical transition of TiO_2_. By comparing the spectra of bare TiO_2_ and of Ag@TiO_2_, it was evident the red shift of the doped sample moved towards the visible region due to the presence of silver ions into the lattice. Notably, the Ag@TiO_2_ spectrum does not show the typical plasmon resonance of silver nanoparticles. This is a further evidence of the successful incorporation of silver ions into the lattice of TiO_2_. The introduction of silver ions, in fact, is reported to introduce intermediate energy states deriving from the strong electronic coupling between the orbitals of TiO_2_ and of silver. In particular, spin polarized density of states analysis revealed strong hybridization between the silver orbitals and the Ti 3d and O 2p bands near the Fermi level [50]. The delocalized electron cloud results in a strong bonding between Ag atoms and the oxygen of TiO_2_. This strongly interacting electronic structure results in a bathochromic shift of the absorption edge, similarly to what reported for strongly interacting components of a solid solution, as in the case of vanadium, chromium, iron, or nickel doped TiO_2_ prepared by ion implantation [51] or for Fe_2_O_3_ heterojunctions with ZnO [16] and TiO_2_ [52]. This behavior significantly differs from the more common weak interaction case, as in the case of silver islands on the surface of TiO_2_, usually generating novel absorption shoulders. According to the Kubelka–Munk theory the band gap energy of bare Ag_3_PO_4_, TiO_2_–Ag_3_PO_4_, and Ag@TiO_2_–Ag_3_PO_4_ was ca. 2.3 eV, while the band gap of TiO_2_ and Ag@TiO_2_ were 3.2 and 3.0 eV, respectively. The narrow band gap of the Ag@TiO_2_ with respect to TiO_2_ makes it a better candidate to be part of a visible light active heterojunction with Ag_3_PO_4_. However, the knowledge of the potential edge of the photogenerated electrons is required to establish the nature of the interfacial electron transfer between the components. To this aim we experimentally determined the quasi Fermi level of both Ag@TiO_2_ and TiO_2_ samples by means of the method proposed by Roy et al. [53]. This method allows one to estimate the quasi Fermi level of a powdered semiconductor in water suspension and under irradiation, i.e., under experimental conditions similar to those used during the photocatalytic experiments. In fact, increasing the pH of the suspension under irradiation shifts cathodically the band edges of the semiconductor, until the one-electron reduction of the electron scavenger is thermodynamically allowed. This occurs at the inflection point of the potentiometric curve, where the quasi Fermi level of electrons equals the pH independent reduction potential of the electron acceptor.

**Figure 5:**
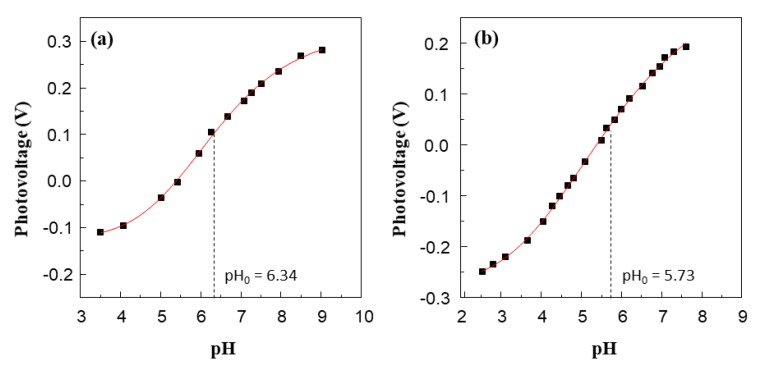
Effect of pH on the photovoltage developed by irradiation of aqueous suspension of TiO_2_ (**a**) and Ag@TiO_2_ (**b**) in the presence of MV^2+^ and DP^2+^, respectively, as electron scavengers. Each experiment has been repeated 3 times and reproducibility was better than ± 0.02 V.

Trials to determine the quasi Fermi level of Ag_3_PO_4_ with this method failed, so that we used the conduction band edge reported in literature (0.45 V vs. NHE at pH 7) [54,55]. Figure 5a,b show the photovoltage vs. pH curves obtained by irradiating TiO_2_ and Ag@TiO_2_ suspensions in the presence of MV^2+^ and DP^2+^, respectively, as the electron acceptors. The use of DP^2+^ instead of MV^2+^, whose structures are shown in the Supporting Information (Figure S6), was necessary because in the presence of MV^2+^ the flex point would have been evident at too high pH values (ca. 9). Notably, the determination of the quasi-Fermi level of electrons of TiO_2_ does not depend on the nature of the electron scavenger used, as demonstrated by Kisch et al. [56].

The pH values of the inflection point (pH_0_) of the obtained sigmoidal titration curves, i.e., pH_0_ equal to 6.34 and 5.73 for TiO_2_ and Ag@TiO_2_ samples, allow one to calculate the flat band potential at pH 7 using Equation (1):

E_FB_ (pH = 7) = E _EA_^2+^_/EA_^+^^∙^ + 0.059 (pH_0_ − 7) (1)

where E _EA_^2+^_/EA_^+∙^ is the standard potential of the electron acceptor used, i.e. MV^2+^/MV^+˙^ equal to −0.45 V vs. NHE and DP^2+^/DP^+˙^ equal to −0.27 V vs. NHE [54]. The resulting potentials of the photogenerated electrons were −0.5 and −0.34 V for TiO_2_ and Ag@TiO_2_, respectively. 

**Figure 6:**
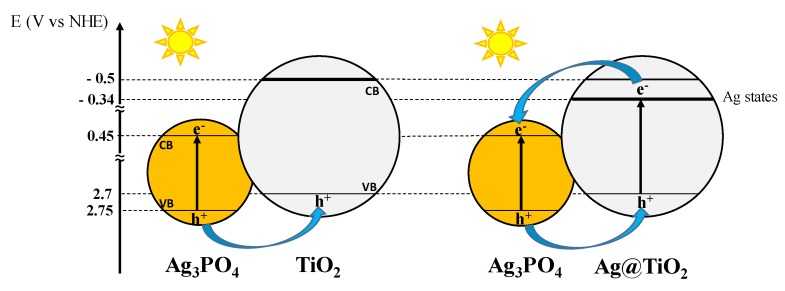
Mechanism of charge separation in the systems TiO_2_–Ag_3_PO_4_ and Ag@TiO_2_–Ag_3_PO_4_. Notably, under solar light irradiation also excitation of bare TiO_2_ take place, even if at lower extent due to the low fraction of UV photons (ca. 5%) in the solar light. For this reason, and for the sake of clarity, Figure 6 does not show this effect.

By taking into account these values, the one reported in literature for Ag_3_PO_4_ [54,55], and the measured band gap energies, the simplified electronic map of the two heterojunctions Ag@TiO_2_–Ag_3_PO_4_ and TiO_2_–Ag_3_PO_4_, shown in Figure 6, can be proposed.

The visible part of the solar spectrum is not able to excite the TiO_2_ counterpart of the TiO_2_–Ag_3_PO_4_ sample, while the presence of substitutional silver within the bulk of Ag@TiO_2_ enables visible light excitation of the Ag@TiO_2_ nanoparticles in the Ag@TiO_2_–Ag_3_PO_4_ sample. In both the composites the photogenerated charges are efficiently separated; however, the excitation of the Ag@TiO_2_ counterpart enables generation of electrons at the silver intermediate energy states from which they can be more efficiently injected into the conduction band of Ag_3_PO_4_.

### 3.3. Photocatalytic Activity under UV and Natural Solar Light Irradiations

**Figure 7:**
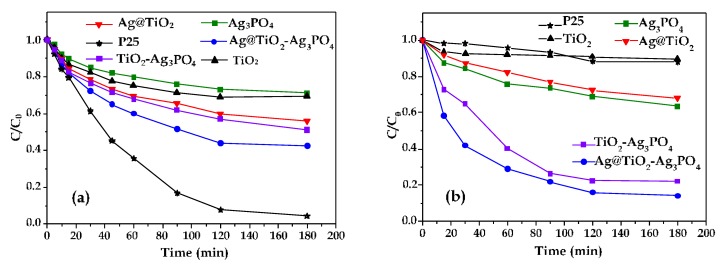
Normalized concentration of 4–NP during the irradiation time (**a**) under UV light and (**b**) under natural solar light of the (■) Ag_3_PO_4_, (▼) Ag@TiO_2_, (▲) TiO_2_, (*) P25, (●) Ag@TiO_2_–Ag_3_PO_4_, and (■) TiO_2_–Ag_3_PO_4_ samples.

Figure 7 shows the photodegradation of the 4–NP under UV light (Figure 7a) and under natural solar light (Figure 7b) in the presence of the investigated photocatalysts. For the sake of comparison, results obtained in the presence of the commercial TiO_2_ P25 Evonik are also reported.

The observed initial rate constants (k_obs_) for each run in Figure 7 have been calculated by differentiating the experimental data at the initial time. The obtained values are reported in Table 2.

Under UV light (Figure 7a), the commercial P25 was the most active sample. The composites, and in particular the Ag@TiO_2_–Ag_3_PO_4_ one, were more active than TiO_2_, Ag_3_PO_4_, and Ag@TiO_2_ samples. Ag_3_PO_4_ showed the lowest activity despite its narrow band gap. The situation dramatically changes under solar light irradiation (Figure 7b), which mainly contains visible light photons, being the UV component only ca. 5%. TiO_2_ and P25 samples were poorly active under solar light, according to their optical properties, while the presence of substitutional silver into the lattice of Ag@TiO_2_ endows it with higher photocatalytic activity, comparable with that of Ag_3_PO_4_. Moreover, the composites, and in particular the Ag@TiO_2_–Ag_3_PO_4_ one, were the most active samples. Notably, Ag doping almost doubles the initial degradation rate in the Ag@TiO_2_–Ag_3_PO_4_ sample with respect to the TiO_2_–Ag_3_PO_4_ one. 

**Figure 8:**
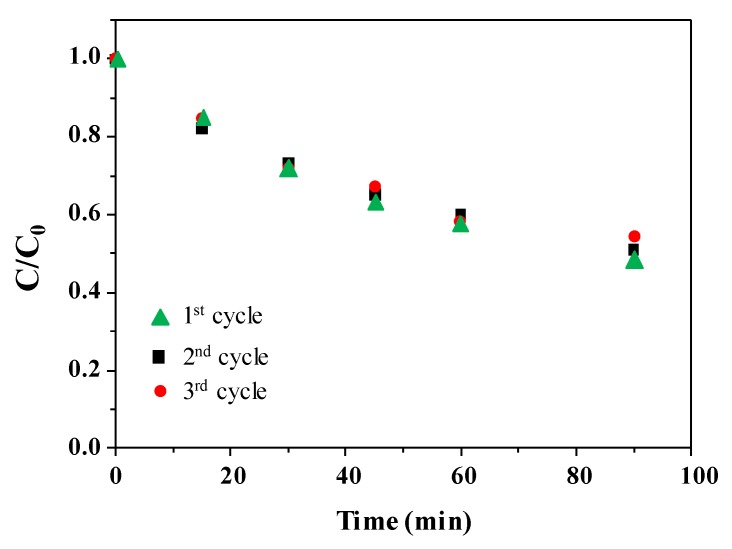
Normalized concentration of 4–NP during the irradiation time for three consecutive photocatalytic runs in the presence of the same Ag@TiO_2_–Ag_3_PO_4_ powder recovered after each cycle.

It is known that coupling Ag_3_PO_4_ with TiO_2_ mitigates the photocorrosion of the Ag_3_PO_4_ counterpart [29]. In order to check if the same holds also for the Ag@TiO_2_–Ag_3_PO_4_ under UV light, three photocatalytic runs were consecutively performed by using the same powder recovered after each cycle. Results are shown in Figure 8.

The superior activity of the Ag@TiO_2_–Ag_3_PO_4_ photocatalyst reflects the above discussed charge transfer mechanism (Figure 6). The existence of heterojunctions between TiO_2_ and Ag_3_PO_4_ has been already reported to enhance photocatalytic activity of the composites with respect to the components. In fact, it is known that the close contact between two semiconductors of suitable electronic structure triggers interfacial electron transfer, effective spatial separation and longer life time of the photogenerated charges, which in turn account for the higher activity. This mechanism can be also invoked in the present case for both the Ag@TiO_2_–Ag_3_PO_4_ and TiO_2_–Ag_3_PO_4_ systems. In fact, the suitable relative position of the valence and conduction bands in both cases allows migration of the holes generated under visible light irradiation in the valence band of Ag_3_PO_4_ to the valence band of TiO_2_. However, in this paper we highlight that silver modification of the TiO_2_ counterpart results in an even higher activity under solar light irradiation. In fact, according to the DRS results, silver doping enables the visible absorption of the Ag@TiO_2_ material, so that under solar light irradiation it is possible to generate higher amount of excitons localized on both Ag@TiO_2_ and Ag_3_PO_4_. Moreover, in this case electrons generated into the conduction band (or into intermediate energy states) of Ag@TiO_2_ can also migrate toward the conduction band of Ag_3_PO_4_ thus enhancing the spatial charge separation. Finally, these effects not only account for the improved photocatalytic activity of the heterojunction, but also for its photo stability. In fact, the improved interfacial electron transfer delocalizes the photogenerated holes also into the valence band of Ag@TiO_2_, thus reducing the reported photocorrosion of Ag_3_PO_4_.

## 4. Conclusions

Heterojunction between silver doped TiO_2_ (Ag@TiO_2_) and Ag_3_PO_4_ has been proved to be a suitable candidate for photocatalytic remediation under solar light irradiation due to its outstanding photocatalytic activity. Physico-chemical characterization of the composites suggests the reasons of the superior performances with respect to the bare components and to the commercial benchmark TiO_2_ P25 (Evonik). The optoelectronic features of the Ag@TiO_2_–Ag_3_PO_4_ sample played a key role and determine its superior performances. In fact, fast charge transfer between the components resulted in efficient charge separation and higher photocatalytic efficiency. In particular, the photocatalytic activity of Ag@TiO_2_–Ag_3_PO_4_ was higher than that of TiO_2_–Ag_3_PO_4_. In fact, silver doping enables visible absorption also of the TiO_2_ counterpart and resulted in the generation of higher amount of excitons localized on both Ag@TiO_2_ and Ag_3_PO_4_ under solar light irradiation. This also improved spatial charge separation with respect to the TiO_2_–Ag_3_PO_4_ sample, because electrons photogenerated in the conduction band of Ag@TiO_2_ could now migrate toward the conduction band of Ag_3_PO_4_. For the outstanding photocatalytic activity highlighted in this work, Ag@TiO_2_–Ag_3_PO_4_ could be proposed for solar light photocatalytic applications. 

## Figures and Tables

**Table 1:** Lattice parameters (a, b, and c), mean crystallites dimension (D), microstrain values (ε), specific surface area (SSA and BET), and pores volume (V_p_) of the samples indicated. Digits under brackets represent relative error influencing the last significant digit of the calculated value.

Samples	a (Å)	b (Å)	c (Å)	D (nm)	ε	SSA (m^2^∙g^−1^)	V_p_ (cm^3^∙g^−1^)
Ag_3_PO_4_	6.0209(1)	6.0209(1)	6.0209(1)	332(8)	-	0.5	0.003
TiO_2_	3.7911(3)	3.791	9.523(1)	26.1(4)	0.00288(3)	80	0.205
Ag@TiO_2_	3.794(1)	3.794	9.499(3)	23.6(8)	0.0058(2)	54	0.109
TiO_2_–Ag_3_PO_4_	-	-	-	-	-	1.9	0.005
Ag@TiO_2_–Ag_3_PO_4_	-	-	-	-	-	1.7	0.004

**Table 2:** Observed initial rate constants (k_obs_ (mg∙L^−1^∙min^−1^)) of 4–NP degradation under UV and natural solar light irradiation in the presence of the considered photocatalytic powders.

k_obs_∙10^2^	Ag_3_PO_4_	TiO_2_	P25	Ag@TiO_2_	TiO_2_–Ag_3_PO_4_	Ag@TiO_2_–Ag_3_PO_4_
UV light	6.8	8.8	15.8	10.4	11.1	12.6
Solar light	6.8	3.8	0.6	5.5	13.5	26.9

## Supplementary Materials

The following are available online at https://www.mdpi.com/2079-4991/10/4/795/s1, Figure S1: XRD patterns of the TiO_2_ sample. Figure S2: XRD patterns of the Ag@TiO_2_ sample. Figure S3: XRD patterns of the Ag_3_PO_4_ sample. Figure S4: XRD patterns of the TiO_2_–Ag_3_PO_4_ sample. Figure S5: EDS analysis of (a) Ag_3_PO_4_, (b,) Ag@TiO_2_ and (c) Ag@TiO_2_–Ag_3_PO_4_ samples. Figure S6: Structures of the electron acceptors DP^2+^ and MV^2+^.
