# Supplementary material for: Enhanced Solar Light Photocatalytic Activity of Ag Doped TiO2–Ag3PO4 Composites
Source: Nanomaterials (Basel). 2020 Apr 21;10(4):795. doi: 10.3390/nano10040795 (PMC7221970; doi:10.3390/nano10040795)
Supplement: Supplementary file 1 [file nanomaterials-10-00795-s001.pdf]

nanomaterials

Supplementary Materials: Enhanced Solar Light Photocatalytic Activity of Ag Doped TiO_2–Ag_3PO_4 Composites

Abdessalem Hamrouni [1], Hanen Azouzi [1], Ali Rayes [1], Leonardo Palmisano [2], Riccardo Ceccato [3] and Francesco Parrino [3,*]

[1] Laboratoire de Recherche Catalyse et Matériaux pour l'Environnement et les Procédés URCMEP (UR11ES85), Faculté des Sciences de Gabès, Université de Gabès, Campus Universitaire Cité Erriadh, Gabès 6072, Tunisia; hamrouni-28@hotmail.fr (A.H.); hanenazzouzi408@gmail.com (H.A.); ali.rayes@fsb.rnu.tn (A.R.)

[2] Department of Engineering, University of Palermo, Viale delle Scienze, Ed. 6, 90128 Palermo, Italy; leonardo.palmisano@unipa.it

[3] Department of Industrial Engineering, University of Trento, Via Sommarive 9, 38123 Trento, Italy; riccardo.ceccato@unitn.it

* Correspondence: francesco.parrino@unitn.it

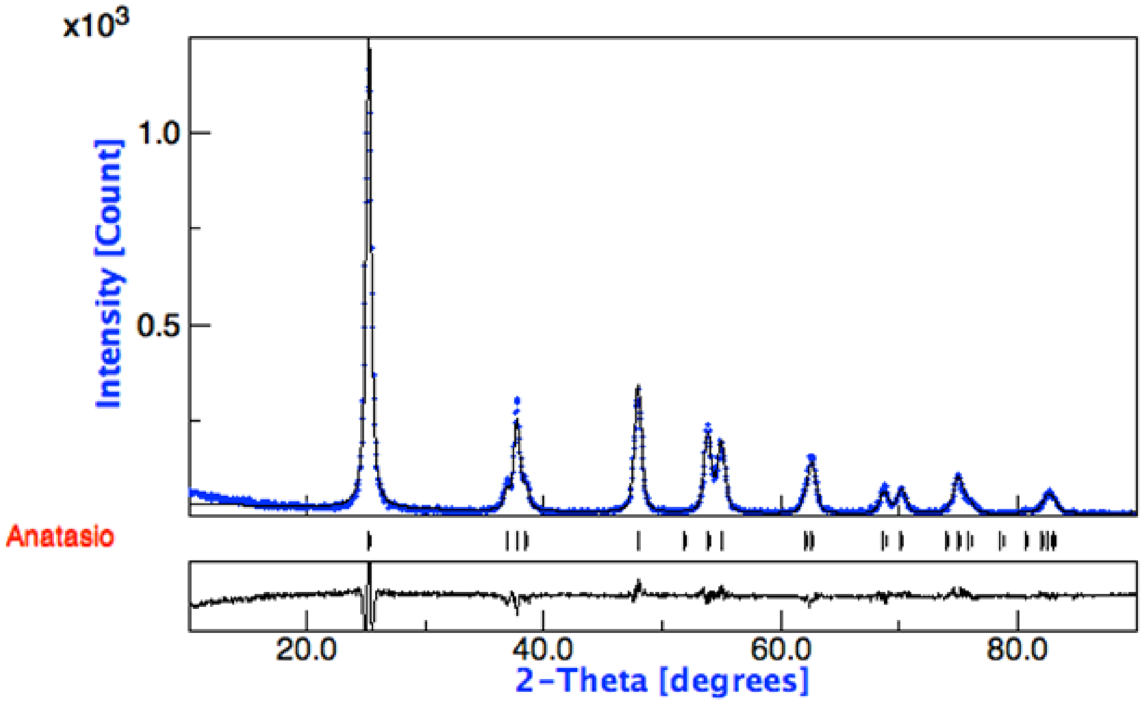

Figure S1. XRD patterns of the TiO_2 sample.

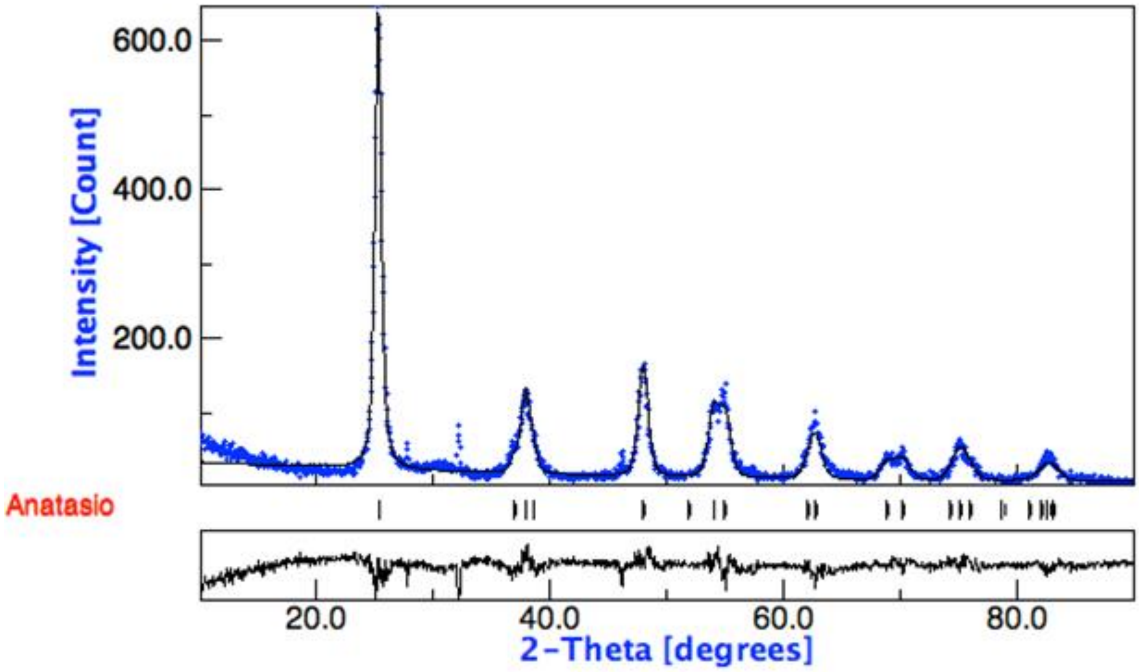

Figure S2. XRD patterns of the Ag@TiO_2 sample.

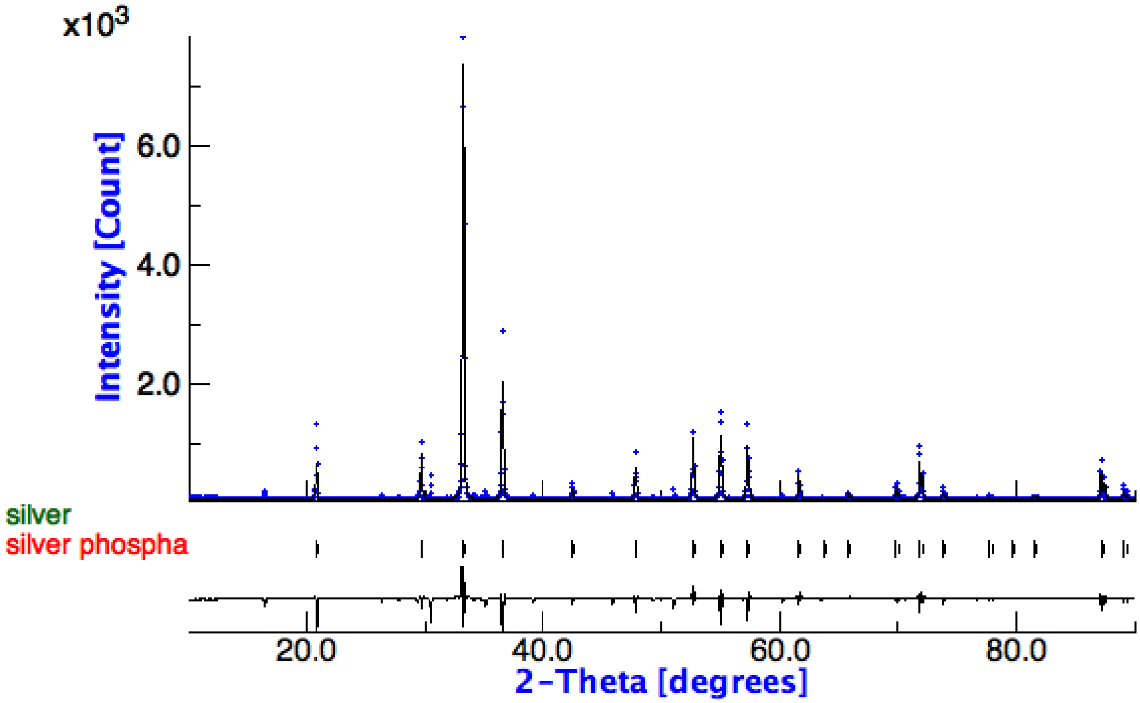

Figure S3. XRD patterns of the Ag_3PO_4 sample.

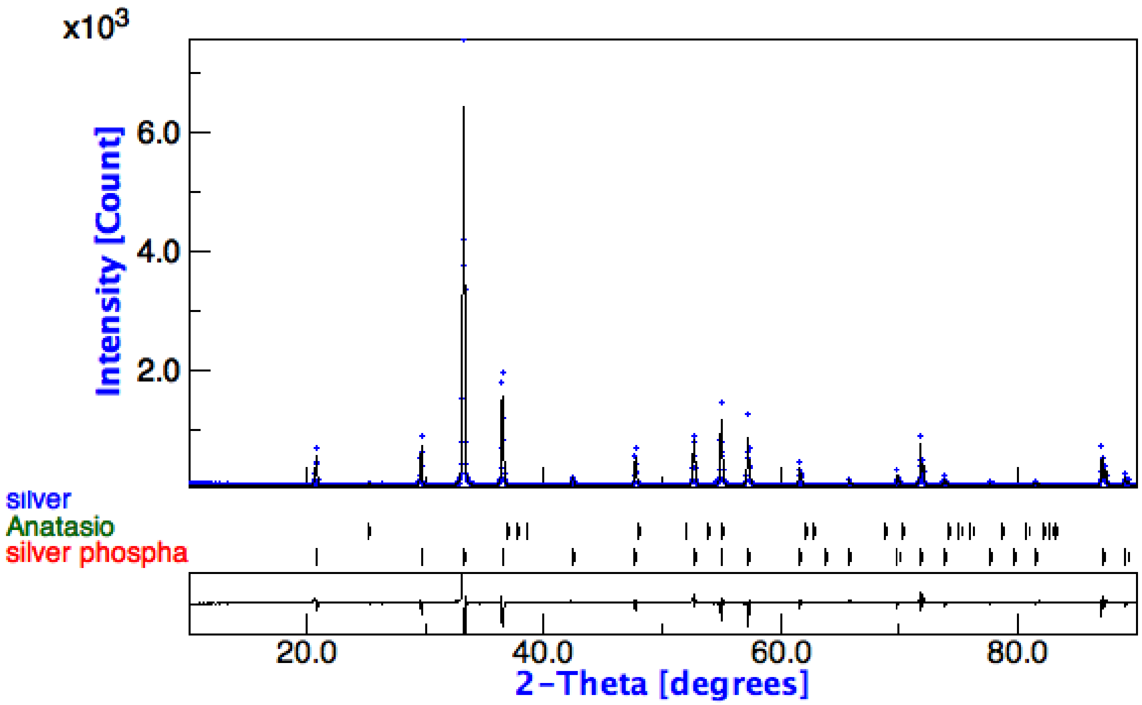

Figure S4. XRD patterns of the TiO_2–Ag_3PO_4 sample.

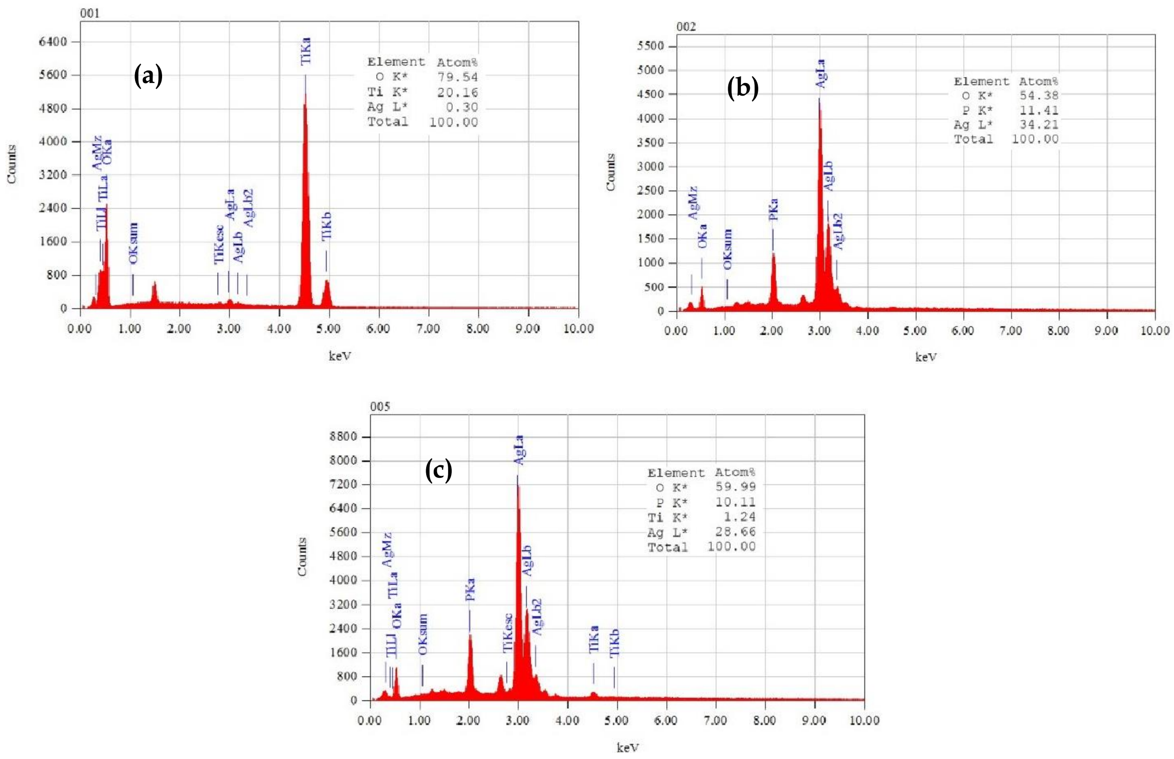

Figure S5. EDS analysis of (**a**) Ag_3PO_4, (**b**) Ag@TiO_2 and (**c**) Ag@TiO_2–Ag_3PO_4 samples.

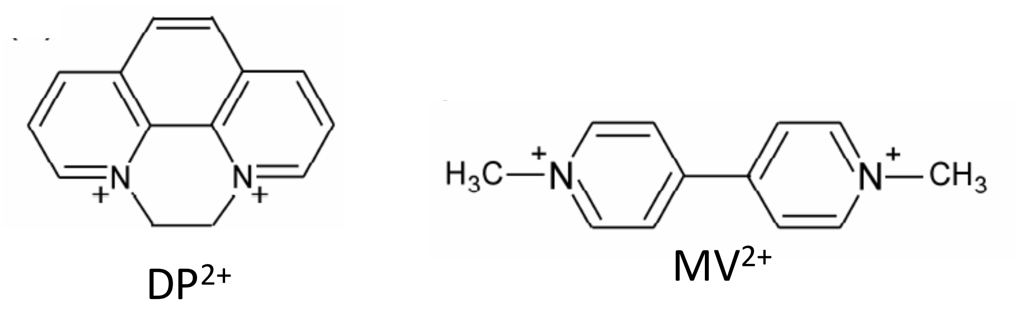

Figure S6. Structures of the electron acceptors DP^{2+} and MV^{2+}.
